# Knowledge, attitudes and practices survey towards pneumococcal infection and vaccination among primary health care physicians, Ukraine, 2021

**DOI:** 10.1371/journal.pone.0304346

**Published:** 2024-06-06

**Authors:** Oksana Artemchuk, Iro Evlampidou

**Affiliations:** 1 Public Health Centre of Ministry of Health of Ukraine, Kyiv, Ukraine; 2 Mediterranean and Black Sea Field Epidemiology Training Programme (MediPIET), Madrid, Spain; Public Library of Science, UNITED KINGDOM

## Abstract

**Background:**

The Ukrainian Ministerial Order (UMO) recommends pneumococcal vaccine (PCV) in risk groups but not free-of-charge resulting in coverage <5% (crude estimation). In 2022, the vaccination calendar will include PCV for children <5years. Doctors’ pneumococcal knowledge, attitudes and practices (КAP) are paramount to successful roll-out but unexplored. We surveyed doctors aiming to assess their KAP to address gaps and misconceptions and support PCV implementation.

**Methods:**

In March 2021, we selected and surveyed primary care doctors using simple random sampling and structured self-administered online questionnaire. We measured attitudes (importance, effectiveness, safety) and practices using 5-point Likert-type questions. We defined pneumococcal disease (PD) knowledge as low/moderate (<80%) and high (≥80%), PCV and overall knowledge as low (≤50%) and moderate/high (51–100%) and PCV attitudes and practices as negative/neutral (1.0–3.4) and positive (3.5–5.0). We calculated prevalence ratios (PRs) and 95% confidence intervals (95%CI) using Poisson regression.

**Results:**

The response rate was 46% (286/628). Females represented 85% (243/285); the median age was 47 (interquartile range: 33–59, N = 281) years. Twenty-six percent (72/277) had high PD knowledge associated with age (>47 years: PR = 0.52, 95%CI: 0.30–0.90) and child-related UMO awareness (PR = 1.78, 95%CI: 1.04–3.08); 65% (182/278) had moderate/high PCV knowledge associated with positive attitudes towards PCV effectiveness (PR = 2.08, 95%CI: 1.20–3.59). Overall knowledge was moderate/high in 69% (188/271); 83% (220/265) had positive PCV attitudes; 52% (135/258) had positive practices associated with female sex (PR = 2.11, 95%CI: 1.09–4.09), positive attitudes (PR = 3.40, 95%CI: 1.23–9.39) and perception of vaccine supply as medium/big barrier (PR = 1.66, 95%CI: 1.02–2.72).

**Conclusion:**

We observed moderate pneumococcal knowledge, especially in older doctors, positive PCV attitudes and neutral practices. Females and doctors with positive attitudes recommended PCV more. For successful PCV implementation, we recommend proper planning and prior educational activities targeting patients and primary care doctors, especially older males, to improve knowledge, introduce PCV and address concerns while ensuring uninterrupted vaccine supply.

## Introduction

Pneumococcal infections, caused by the bacterium *Streptococcus Pneumonia*, are a major public health problem causing, among others, respiratory infections, meningitis and bacteraemia mainly in young children and the elderly while an effective treatment and vaccine exist. According to the World Health Organization (WHO), in 2015, of the 5.83 million estimated global deaths among children <5 years, 294,000 were estimated to be caused by pneumococcal infections [1]. Prior to the introduction of pneumococcal conjugate vaccines (PCV) in the different WHO regions, 6–11 serotypes accounted for ≥70% of all invasive pneumococcal disease in infants and children under 5 years of age.

The WHO recommends the inclusion of PCV in the national childhood immunization programmes worldwide and the USA Centres for Disease Control and Prevention recommends pneumococcal vaccination for all children younger than 2 years old and all adults 65 years and older [2]. For administration of PCV to infants, WHO recommends a 3-dose schedule administered either as 2p+1 schedule (2 doses 8 weeks apart and a booster dose between 9 and 15 months) or as 3p+0 schedule (3 doses 4 weeks apart at 6, 10 and 14 weeks of age) [3].

A study in 2015, reported that 8 years of PCV use in England and Wales reduced the overall incidence of invasive pneumococcal disease by more than 50% [4]. Currently, four types of vaccines are available, PCV-7, PCV-10, PCV-13 and PPSV covering 7, 10, 13 and 23 serotypes, respectively [5]. Also, two additional higher-valency vaccines are available in certain countries for use in adults (PCV15 and PCV20) [6].

In 2019, Ukraine, a country of 38 million population, registered only 50 cases of pneumococcal meningitis, of which 22 in children and in 2018, registered 43 cases, 10 of them in children, while data on other pneumococcal diseases (PD) are not routinely available [7]. Vaccination against pneumococcal infections is only recommended for children <18 years and adults and particularly for children from closed groups (boarding schools, orphanages) and the elderly (≥65 years), especially those living in long term care facilities [8]. However, the PCV is not free nor is reimbursed. In Ukraine, the approximate cost of one dose of vaccine PREVENAR^®^ 13: pneumococcal 13-valent adsorbed polysaccharide conjugate vaccine [diphtheria CRM197 protein]; Wyeth Pharmaceuticals Inc., New Lane, Havant, PO9 2NG, United Kingdom is 75 US dollars. Primary care doctors in state and private health clinics that have a contract with the Ministry of Health (MoH) of Ukraine to perform vaccinations are responsible for the prescription and administration of the PCV vaccine in accordance with the instructions for use.

The vaccination coverage remains low, as of 1^st^ May 2020, 1135 people were vaccinated against pneumococcal infection and 552 were revaccinated [9]. No information is available on the age group of vaccinated individuals, the type of vaccine product used for vaccination and number of vaccine doses revaccinated people received. The Ukrainian Ministerial Order does not contain specific details on the formulations and on the specific vaccine schedule.

In June 2020, the National Technical Group of Immunization Experts, recommended to the MoH to consider the introduction of PCV into the National Vaccination Calendar as mandatory and free of charge for children up to 5 years in 2022 [10].

Various studies have shown that, physicians’ knowledge and positive attitudes towards PCV vaccination is related to positive practices and may affect decisions to strongly recommend and prescribe the vaccine [11–13]. The most commonly reported barriers were patients’ refusal, vaccine cost and physicians’ lack of awareness and knowledge about the vaccine and concerns about its efficacy. In Ukraine, no studies have been previously conducted on the knowledge, attitudes and practices (KAP) of primary care doctors towards pneumococcal diseases and vaccination that remain unknown and unexplored.

The aim of this study was to assess the knowledge of Ukrainian primary care doctors on pneumococcal diseases and vaccination, their attitudes and practices regarding PCV vaccination and to identify possible barriers to the implementation of PCV vaccination in order to guide programmatic decisions and future implementation of PCV in Ukraine.

## Methods

### Study design, area and population

In March 2021, we conducted a nationwide cross-sectional study in Ukraine. The study population was primary healthcare doctors working in Ukraine and specifically, internal medicine doctors, pediatricians and general practitioners working in state or private primary healthcare institutions or private practices and with a contract with the National Health Service of Ukraine to conduct preventive vaccinations. We excluded doctors if their name was not stated in the sampling frame list we used during sampling or if they refused to participate in the study.

#### Sample size

We used simple random sampling to select the study participants, a methodology in which each member of the study population has an equal chance of being invited to participate in our study. As sampling frame, we used a list regularly updated by the MoH of all doctors working in all primary health care institutions that have a contract with the National Health Service of Ukraine for conducting preventive vaccinations. The list is publicly available from the National Health Service of Ukraine and contains information on primary healthcare facility (region, facility name, place, email and postal address) and doctors’ characteristics (name, specialization, telephone(s), number of patients the doctor serves).In the initial list of 29,765 entries, we applied the inclusion and exclusion criteria and excluded 5056 entries without the doctor’s name and 582 duplicate entries. In the final sampling frame list, 24,127 unique observations remained.

A sample of 628 participants (population size: 24,127) was required to obtain prevalence estimates of pneumococcal infection and PCV knowledge of 50% with 5% absolute precision, a 95% confidence level, design effect of 1 and response rate of 30%. The sample size was calculated using the statistical calculator OpenEpi [14].

#### Data collection

We developed a structured self-administered online questionnaire using the Google Forms platform that consisted of 6 sections with 37 questions (yes/no, multiple choice and grid, check-boxes and grid, 5-point Likert-type, and open-ended) (S1 File). The first section consisting of 13 questions included socio-demographic (sex, age, region, specialization, etc.) and workplace (region, facility type, doctors’ and facility practice size, etc.) characteristics. The second section consisted of 6 questions on knowledge of disease (cause, transmission, risk groups, manifestations, severity, and treatment). The third section consisted of 5 questions on knowledge of vaccine (existence, vaccine types, indicated patient groups and number of doses, side effects). The fourth consisted of 3 questions on doctors’ attitudes towards PCV vaccine (importance, effectiveness, safety) and the fifth section consisted of 2 questions on PCV practices (recommendation to children and elderly) and 1 question on the reasons for not recommending the vaccine. Finally, the sixth section included 5 questions about doctors’ perception of 17 possible barriers in PCV implementation (“not a problem”, “small”, “medium” and “big”), possible actions to help in the PCV implementation and about sources, types and ways of receiving information.

The questionnaire was initially developed in the English language and was then translated into Ukrainian by the principal investigator, whose mother tongue is Ukrainian and was reviewed by another team member and a professional translator to ensure the quality of the translation. Before full deployment, we piloted the questionnaire among 3 doctors working in primary healthcare institutions in Kyiv to ensure comprehension and usability of the questions and to define the time needed to complete the survey, and made necessary modifications afterwards (i.e. rephrasing of questions and answers and shortening the questionnaire’s length).

### Data collection procedure

We sent an official letter/invitation with the survey link to the regional state healthcare departments’ directors that they forwarded it to specified healthcare facilities’ directors which in turn sent it to the selected study participants. We used this method because the doctors’ personal emails were not included in the sampling frame list and the primary healthcare facilities are subordinated to the regional state healthcare departments. The invitation was accompanied by an information sheet explaining the study objectives, data use, confidentiality, anonymity, data protection issues, ethics approval and the link to the questionnaire. We sent the invitation in the fourth week of March 2021 allowing one week for the survey completion followed by one reminder after one week. In total, the survey was open for two weeks.

Participants provided their responses anonymously via the online Google Forms platform.

#### Data analysis

We defined as outcome variables participants’ 1) PD knowledge 2) PCV knowledge 3) overall knowledge (disease and vaccine) 4) attitudes towards PCV and 5) practices regarding PCV vaccination.

#### Scores generation

For each outcome we first created continues scores based on the individual answers in each of the respective section’s questions that we then transformed into ordinal and binary for use in subsequent analysis.

For the outcomes of disease and vaccine knowledge, in the multiple-choice questions we gave 1 point for the correct answer, 0 points for the incorrect and we transformed into missing the “I am not sure”–except in the questions on existence of treatment and vaccine and number of doses according to the age-group that we assigned a 0 point. In the questions with multiple correct answers we gave 1 point for each correct and -1 point for each incorrect answer. In the 5-point Likert-type question on disease severity, we gave 1 point for the “very” and “extremely” serious answers and 0 points for the others, and in the question on frequency of PCV side effects, we gave 1 point for the “not frequent at all” and 0 points for the others.

As the course of pneumococcal infection can become severe (pneumococcal blood infection, pneumococcal meningitis) and could lead to death, and pneumococcal infection of ear could affect hearing and cause speech and learning difficulties, we considered very/extremely serious answers as “correct” and moderately/slightly/not at all serious answers as “incorrect”. We therefore gave “incorrect” answer 0 points, while 1 point was awarded for a correct answer as was done in other studies [15–17] that studied knowledge/attitudes towards disease or vaccine.

We considered the “I am not sure” answer for the existence of treatment and vaccine and dosing regimen questions as incorrect as opposed to all questions related to outcomes of pneumococcal disease because we believe it is inappropriate for doctors to reply “not sure” about the existence of treatment and of vaccine and dosing regimen. Treatment of the pneumococcal—a bacterial—infection and of prevention methods (i.e., vaccination) is a part of the doctors’ education in medical schools and therefore they tought to be aware about the fact that treatment and vaccine for pneumococcal infection exist.

Although PCV vaccine is not mandatory vaccination in Ukraine and it is not included into National Immunization Calendar, however, this vaccine belongs to the recommended by Ukraine MoH vaccines and doctors who have a contract with the MoH (i.e. our study participants) to perform vaccinations should be aware how to administer the PCV vaccine.

We created continuous scores by summing the points of each respective section’s related questions: for disease knowledge we included the six PD questions (theoretical range: -7–11) and for PCV knowledge we included the five PCV questions (theoretical range: -8–13). We defined overall knowledge as the sum of the PD and PCV knowledge scores (theoretical range: -15–24). We transformed the continuous scores of PD, PCV and overall knowledge into ordinal and defined them as low if participants scored ≤50% of correct answers, moderate if they scored 51–79% and high if they scored ≥80%. Finally, we created binary variables in which we defined PD knowledge as low/moderate and high and PCV and overall knowledge as low and moderate/high.

The reasoning for categorizing PD knowledge as low/moderate versus high, while PCV knowledge as low versus moderate/high was both context-related and due to statistical reasons. We considered that doctors should be more knowledgeable about the disease since they were taught about it during their studies/specialization comparing to the knowledge of the details of the vaccine that is currently not part of the immunization schedule in Ukraine and hence the doctors might not be very familiar with the details of the vaccine. Additionally, high knowledge of vaccine was very low (4.55%) and could not stand alone as a separate category in the multivariable analysis. We had initially created a binary with PCV knowledge low/moderate vs. high but the models did not run and opted for the low vs. moderate/high categorization.

For attitudes and practices, we used the Likert’s method of Summated Ratings and created a score containing the mean of the 3 attitude questions and another containing the mean of the 2 practices questions (range: 1–5). Lastly, we created binary variables in which we defined PCV attitudes and practices as negative/neutral if the mean was from 1.0–3.4 and as positive if the mean was from 3.5–5.0. For the PCV safety variable, we additionally created a binary variable by combining the answers “Not safe at all”, “Slightly safe”, “Moderately safe” and “Depends on the producer country” into one category and the “Very safe” and “Extremely safe” into another.

For the barriers variables, we created binary variables by combining the answers “Not at all problem” and “Small” into one category and the “Big” and “Medium” into another.

#### Statistical analysis

We performed descriptive statistics and calculated for continuous variables the mean and standard deviation (SD) or the median and interquartile range (IQR) after assessing for normality using the Shapiro-Wilk test, and for categorical variables counts (n), proportions (%) and 95% confidence intervals (95%CI). We explored the association among explanatory variables and between explanatory and outcome variables using for continuous variables the t-test or Wilcoxon ranksum test and for categorical variables Chi-square or Fisher’s exact tests. A significance level of 0.05 was used throughout.

We performed univariate and multivariate Poisson regression analysis for each of the 5 outcomes using the binary variables to identify independent predictors (i.e. explanatory variables) after checking for correlations between explanatory variables using the Pearson’s correlation test, and calculated Prevalence Ratios (PR) and 95%CIs. All explanatory variables that had a p-value of less than 0.150 were included in the initial models. We used stepwise backwards elimination using the Akaike Information Criterion (AIC) and Bayesian Information Criterion (BIC) to reach to the most parsimonious model considering the one with the smallest AIC and/or BIC as the best fitted model.

We extracted data from Google Forms to Microsoft Excel and analysed them in STATA 16 (StataCorp LLC, USA).

#### Ethical approval

This study was approved by the Ethical Committee of the Public Health Centre of the Ministry of Health of Ukraine with Registration number: # 000l1557 and Identification number: IRB2021–55.

An information sheet on the study was provided to the study participants together with the invitation to participate. At the beginning of the online questionnaire, there was a specific mandatory question where participants were asked to acknowledge that they have been informed for the purposes of this study and that they consent to participate. If participants replied “No” to this first consent question, they were automatically transferred to the end of the survey without having to complete the questionnaire since consent was not obtained. Our study did not include minors; therefore, we did not need to obtain consent from parents or guardians.

## Results

### Socio-demographic and workplace characteristics

We invited 628 primary healthcare doctors and 286 responded to the questionnaire (response rate: 46%) among which 243 (85%) were females. Among 281 participants, the median age was 47 (IQR: 33–59; range: 25–80) years and, 78 (28%) had 0–8 years of professional experience while 227 (80%, N = 285) reported the presence of professional category (Table 1). Of 284 participants, 192 (68%) were family doctors, 61 (21%) paediatricians and 31 (11%) internal medicine doctors, 101 (36%) were living in the western part of Ukraine, 199 (70%) were working in urban areas and 107 (38%, N = 283) served 951–1810 patients. Among 285 participants, 272 (95%) worked at public facilities, 145 (51%) at ambulatory clinics and 105 (37) at healthcare centres while 132 (46%) of the facilities had catchment population of >19000 patients.

**Table 1:** Socio-demographic and primary healthcare facilities characteristics, Ukraine, 2021.

Characteristics	n	%	95%CIs^a^
**Sex: Female (N = 285)**	243	85	81–89
**Age group (years) (N = 281)**			
25–33	75	27	22–32
34–47	67	24	19–29
48–59	69	25	20–30
59–80	70	25	20–30
**Professional experience (years) (N = 281)**			
0–8	78	28	23–33
9–20	65	23	18–29
21–34	69	25	20–30
>34	69	25	20–30
**Presence of professional category (N = 285)**	227	80	75–84
**Doctors’ specialization (N = 284)**			
Family doctor	192	68	62–73
Paediatrician	61	21	17–27
Internal medicine	31	11	7.5–15
**Area of work (N = 284)**			
West	101	36	30–41
North	67	24	19–29
South	50	18	13–23
Centre	35	12	8.7–17
East	31	11	7.5–15
**Place of work (N = 284)**			
Urban	199	70	64–75
Rural	85	30	25–36
**Size of doctor’s practice (N = 283)**			
<950	91	32	27–38
951–1810	107	38	30–42
>1810	85	30	25–36
**Health care facility sector (N = 285)**			
Public	272	95	92–98
Private	13	4.6	2.5–7.7
**Health care facility type (N = 286)**			
Ambulatory	145	51	45–57
Health centre	105	37	31–43
Polyclinic	25	8.7	5.7–13
Private	11	3.9	1.9–6.8
**Catchment population (N = 285)**			
< = 3000	39	14	9.9–18
3001–19000	114	40	34–46
>19000	132	46	40–52

^a^ 95% confidence intervals

#### Pneumococcal disease and vaccine knowledge

Among 285 study participants, 277 (97%) were aware that PD is a bacterial infection, 246 (88%, N = 280) that is transmitted via person-to-person droplets, while 199 (70%, N = 286) indicated the most affected patient groups as children up to 5 years and 126 (44%, N = 286) as the elderly (≥65 years) (Table 2). Also, 215 (76%, N = 284) participants stated that a treatment exists and 268 (94%, N = 285) that the clinical course can become extremely/very severe. Regarding PD clinical manifestations, among 286 respondents, 274 (96%) correctly identified pneumonia, 181 (63%) meningitis, 173 (60%) otitis, 131 (46%) bacteraemia and 108 (38%) sinusitis.

**Table 2:** Knowledge towards pneumococcal disease and vaccine (PCV) and respective scores among primary care doctors, Ukraine, 2021.

	n	%	95%CIs^a^
***Pneumococcal disease***			
**Aetiology (N = 285)**			
Bacterium	277	97	95–99
Other (virus, fungus)	8	2.8	1.2–5.5
**Affected patients groups (N = 286)** ^b^			
Children up to 5 years	199	70	64–75
Elderly 65 years and older	126	44	38–50
Patients with comorbidities	92	32	27–38
Children of all age-groups	59	21	16–26
Adults of all age-groups	53	19	14–24
**Transmission (N = 280)**			
Person-to-person droplets	246	88	83–91
Direct contact	34	12	8.6–17
**Clinical manifestations (N = 286)** ^b^			
Pneumonia	274	96	93–98
Meningitis	181	63	57–69
Otitis	173	60	55–66
Bacteraemia	131	46	40–52
Sinusitis	108	38	32–44
Others (urinary infection, deterioration of vision)	16	5.6	3.2–8.9
**Severity (N = 285)**			
Very/extreme	268	94	91–96
Slight/moderate	17	6.0	3.5–9.4
**Existence of treatment: yes (N = 284)**	215	76	70–81
***Pneumococcal vaccine***			
**Existence of vaccine: yes (N = 286)**	274	96	93–98
**Types of PCV vaccine (N = 271)** ^b^			
PCV13	242	89	85–93
PPSV23	92	34	28–40
PCV 7	52	19	15–24
Others (PCV20, PCV 40)/Don’t know	40	15	11–20
**Number of PCV doses according to age (N = 274)** ^b^			
Children 2–6 months, 2 doses^c^	173	63	57–69
Children 7–23 months, 2 doses	213	78	72–83
Children 24–59 months, 1 dose	140	51	45–57
Elderly ≥65years, 1 dose	177	65	59–70
**PCV side effects (N = 272)**			
Not frequent	218	80	75–85
Moderately frequent	35	13	9.1–17
Frequent	19	7.0	4.3–11
**PCV recommendations according to the UMO (N = 272)** ^b^			
Elderly (≥65 years), especially living in long care facilities	183	67	61–73
Children—all ages	160	59	53–65
Children in closed groups (boarding schools, orphanages)	141	52	46–58
Adults—all ages	56	21	16–26
Others (lactating women, teenagers 13–16 years, comorbidities)	125	46	40–52
I am not aware of the ministerial order	6	2.2	0.8–4.7
***Knowledge scores***			
**Pneumococcal disease (N = 277)**			
Low/moderate (0–79%)	205	74	68–79
High (80–100%)	72	26	21–32
**Pneumococcal vaccine (N = 278)**			
Low (0–50%)	96	35	29–40
Moderate/high (51–100%)	182	65	60–71
**Overall (N = 271)**			
Low (0–50%)	83	31	25–36
Moderate/high (51–100%)	188	69	64–75

UMO: Ukrainian Ministerial Order

^a^ 95% confidence intervals

^b^ Numbers don’t add up to 100% because of multiple answers

^c^ children 2–6 months, 2 doses: in the online questionnaire, we mistakenly omitted the option “3 doses” for this question; therefore, we considered the answer of two doses as the correct one for this age group in subsequent analysis

The median score for PD knowledge was 7 (IQR 5–9; range: 2–11) with 205 (74%, N = 277) participants demonstrating low/moderate knowledge. High PD knowledge was positively associated with awareness of child-related Ukrainian ministerial order (UMO) (PR = 1.78, 95%CI: 1.04–3.08) and negatively with age >47 years (PR = 0.52, 95%CI: 0.30–0.90) (Table 3).

**Table 3:** Factors associated with high pneumococcal disease knowledge among primary care doctors, Ukraine, 2021.

High disease knowledge (N = 72, 26%) (vs. low/moderate)	n	Total	%	aPR^a^	95%CIs^b^
**Child-related UMO** ^c^					
No	22	127	17	ref.	
Yes	48	138	35	1.78	1.04–3.08
**Age (years)**					
≤47	50	137	37	ref.	
>47	20	135	15	0.52	0.30–0.90
**Vaccine safety**					
Low/moderate	20	106	19	ref.	
High	46	141	33	1.41	0.82–2.45

UMO: Ukrainian Ministerial Order

^a^ Adjusted Prevalence ratio using Poisson regression

^b^ 95% confidence intervals

^c^ Knowledge that the UMO recommends the pneumococcal vaccine to all children

Among all participants, 274 (96%) stated that the PCV exists and among 271, 242 (89%) identified the PCV13, 92 (34%) the PPSV23 and 52 (19%) the PCV7 as the types of vaccine that are currently available (Table 2). Among 274 participants that reported the number of PCV doses needed according to age, 173 (63%) indicated 2 doses for children 2–6 months, 213 (78%) 2 doses for children 7–23 months, 140 (51%) 1 dose for children 24–59 months, and 177 (65%) 1 dose for elderly ≥65years. Of 272 participants, 218 (80%) stated that PCV side effects are not frequent at all while 183 (67%) identified the elderly (≥65 years), especially living in long care facilities and 160 (59%) identified children of all ages as patient groups recommended to vaccinate in the UMO.

The median score for PCV knowledge was 8 (IQR: 6–9; range: 0–12) with 182 (65%, N = 278) participants demonstrating moderate/high knowledge. Moderate/high PCV knowledge was associated with positive attitudes towards PCV effectiveness (PR = 2.08, 95%CI: 1.20–3.59) (Table 4).

**Table 4:** Factors associated with high pneumococcal vaccine (PCV) knowledge among primary care doctors, Ukraine, 2021.

Moderate/high PCV knowledge (N = 182, 65%) (vs. low)	n	Total	%	aPR^a^	95%CIs^b^
**Attitudes towards PCV effectiveness**					
Low/moderate	16	42	38	ref.	
High	166	223	74	2.08	1.20–3.59
**Pneumococcal disease knowledge**					
Low/moderate (0–79%)	120	201	60	ref.	
High (80–100%)	59	70	84	1.26	0.91–1.74
**Age (years)**					
≤47	101	138	73	ref.	
>47	76	135	56	0.86	0.63–1.17

^a^ Adjusted Prevalence ratio using Poisson regression

^b^ 95% confidence intervals

The median score for overall pneumococcal knowledge was 15 (IQR: 12–18; range: 2–23) with 188 (69%, N = 271) participants demonstrating moderate/high knowledge (Table 2). We did not identify any factors to be associated with moderate/high overall knowledge in the multivariate analysis (Table 5).

**Table 5:** Factors associated with moderate/high overall pneumococcal knowledge among primary care doctors, Ukraine, 2021.

Moderate/high overall knowledge (N = 188, 69%) (vs. low)	n	Total	%	aPR^a^	95%CIs^b^
**Vaccine safety**					
Low/moderate	69	104	66	ref.	
High	111	137	81	1.15	0.84–1.57
**Age (years)**					
≤47	110	135	81	ref.	
>47	74	131	56	0.76	0.56–1.04

^a^ Adjusted Prevalence ratio using Poisson regression

^b^ 95% confidence intervals

#### PCV attitudes

Among participants, 209 (76%, N = 274) believed that PCV is very/extremely important against protection from pneumococcal disease in at-risk patients, 229 (84%, N = 272) that is very/extremely effective in preventing invasive disease and 147 (58%, N = 253) that is very/extremely safe (Table 6).

**Table 6:** Attitudes towards pneumococcal vaccine (PCV) and score among primary care doctors, Ukraine, 2021.

*Attitudes*	n	%	95%CIs^a^
**PCV importance (N = 274)**			
High	209	76	71–81
Moderate	42	15	11–20
Low	23	8	5.4–12
**PCV effectiveness (N = 272)**			
High	229	84	79–88
Moderate	35	13	9.1–17
Low	8	2.9	1.3–5.7
**PCV safety(N = 253)**			
High	147	58	52–64
Moderate	48	19	14–24
Depends on the producer country	44	17	13–23
Low	14	5.5	3.1–9.1
***Attitudes Score (N = 265)***			
Positive (3.5–5.0)	220	83	78–87
Negative/neutral (1.0–3.4)	45	17	13–22

^a^ 95% confidence interval

The mean score for PCV attitudes was 4.3 (IQR: 3.6–4.5; range: 2–5) with 220 (83%, N = 265) participants demonstrating positive attitudes. We did not identify any factors associated with positive attitudes in the multivariate analysis (Table 7).

**Table 7:** Factors associated with positive attitudes towards pneumococcal vaccine (PCV) among primary care doctors, Ukraine, 2021.

Positive attitudes (N = 220, 83%) (vs. negative/neutral)	n	Total	%	aPR^a^	95%CIs^b^
**Practice towards PCV**					
Negative (1.0–2.6)	45	68	66	ref.	
Neutral (2.7–3.4)	38	48	79	1.17	0.75–1.82
Positive (3.4–5.0)	126	134	94	1.41	1.00–2.00
**Overall knowledge**					
Low (0–50%)	54	69	78	ref.	
Moderate/high (51–100%)	155	182	85	1.11	0.78–1.56
**Age (years)**					
≤47	112	137	82	ref.	
>47	103	123	84	1.05	0.78–1.41

^a^ Adjusted Prevalence ratio using Poisson regression

^b^ 95% confidence intervals

#### PCV practices

Participants often/always recommended PCV to children (132/222; 59%) and to the elderly (82/211; 39%) (Table 8). The most frequent reasons not to recommend PCV to children (N = 90) and to the elderly (N = 143) were the vaccine’s high cost (children: n = 49; 54%, elderly: n = 83; 58%), patient’s mistrust to PCV (children: 34, 38%; elderly: 41, 29%) and patients’ refusal of any vaccinations (children: 10, 11%; elderly: 25, 18%).

**Table 8:** Practices towards pneumococcal vaccine (PCV) and reasons for not recommending it to patient groups among primary care doctors, Ukraine, 2021.

	Children			Elderly		
	n	%	95%CIs^a^	n	%	95%CIs^a^
**PCV recommendation to children (N = 222) and elderly (N = 211)**						
Often/always	132	59	53–66	82	39	32–46
Rarely/sometimes	63	28	23–35	108	51	44–58
Never	27	12	8.2–17	21	10	6.2–15
**Reasons for not recommending PCV to children (N = 90) and elderly (N = 143)**						
The vaccine is too expensive for my patients	49	54	44–65	83	58	50–66
My patients have a mistrust to PCV	34	38	28–49	41	29	21–37
My patients refuse any vaccinations	10	11	5.5–19	25	18	12–25
PCV is not an important vaccination for this patient group	6	6.7	2.5–14	14	9.8	5.5–16
I do not think PCV vaccine is effective	2	2.2	0.3–7.8	2	1.4	0.2–5.0
I do not think PCV vaccine is safe	0	0.0	0.0–0.0	1	0.7	0.0–3.8
Other	14	16	8.8–25	18	13	7.6–19

^a^ 95% confidence intervals

The mean score of PCV practices was 3.3 (SD: 1.1; range: 1–5) with 135 (52%, N = 258) participants obtaining positive scores. Positive PCV practices were associated with positive attitudes (PR = 3.40, 95%CI: 1.23–9.39), female sex (PR = 2.11, 95%CI: 1.09–4.09) and identification of vaccine unavailability as medium/big barrier (PR = 1.66, 95%CI: 1.02–2.72) (Table 9).

**Table 9:** Factors associated with positive pneumococcal vaccine (PCV) practices among primary care doctors, Ukraine, 2021.

Positive vaccine practices (N = 135, 52%) (vs. negative/neutral)	n	Total	%	aPR^a^	95%CIs^b^
**PCV attitudes**					
Negative/neutral (1.0–3.4)	8	41	20	ref.	
Positive (3.5–5.0)	126	209	60	3.40	1.23–9.39
**Sex**					
Male	13	36	36	ref.	
Female	121	221	55	2.11	1.09–4.09
**Barrier: vaccine unavailability**					
No/small	31	76	41	ref.	
Medium/big	93	153	61	1.66	1.02–2.72
**Satisfaction with the MoH webpage about PCV**					
Low/moderate	66	138	48	ref.	
High	62	96	65	1.41	0.94–2.11
**Barrier: Lack of staff**					
No/small	100	194	52	ref.	
Medium/big	26	43	60	1.23	0.70–2.17
**Age (years)**					
≤47	66	132	50	ref.	
>47	66	121	55	1.16	0.75–1.79
**Overall knowledge**					
Low (0–50)	30	66	45	ref.	
Moderate/high (51–100%)	94	179	53	0.98	0.60–1.61
**Barrier: Unknown prior patient’s immunization status**					
No/small	88	149	59	ref.	
Medium/big	36	81	44	0.83	0.50–1.37

MoH: Ministry of Health of Ukraine

^a^ Adjusted Prevalence ratio using Poisson regression

^b^ 95% confidence intervals

#### Perceived barriers and possible actions in PCV implementation

**Fig 1:**
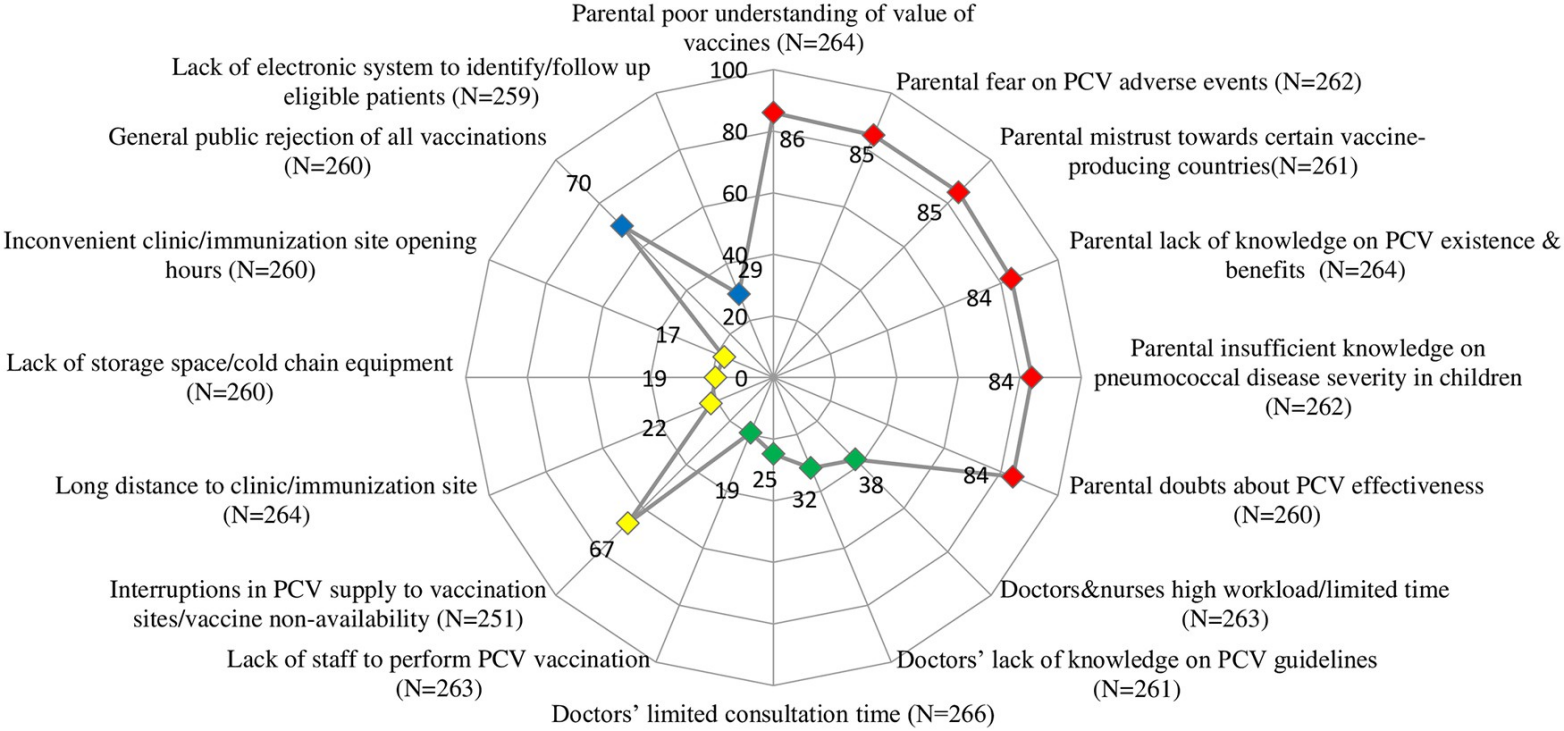
Perception of barriers towards pneumococcal vaccine (PCV) implementation as big or medium among primary care doctors, Ukraine, 2021. Red markers depict parental related barriers; green markers depict doctors’ and nurses’ related barriers; yellow markers depict logistics and access related barriers; blue markers depict other types of barriers.

Participants reported mainly parental-related issues as medium/big problems in the implementation of PCV in children up to 5 years in Ukraine including poor understanding of the real value of vaccines in general (227/264; 86%), fear on the possible PCV adverse events (223/262; 85%) and mistrust towards certain vaccine-producing countries (222/261; 85%) (Fig 1). Parental-related barriers were significantly correlated to each other (Pearson’s correlation coefficient p-values<0.001). Doctors/nurses’ related barriers and logistic related issues as medium/big problems were reported less. For example, participants evaluated as medium/big problems the high workload of doctors and nurses and limited time to perform PCV vaccination (99/263; 38%) and interruptions in the PCV supply to vaccination sites (168/251; 67%). Doctors’/nurses’-related barriers were also highly correlated to each other (Pearson’s correlation coefficient p-values = 0.000).

**Fig 2:**
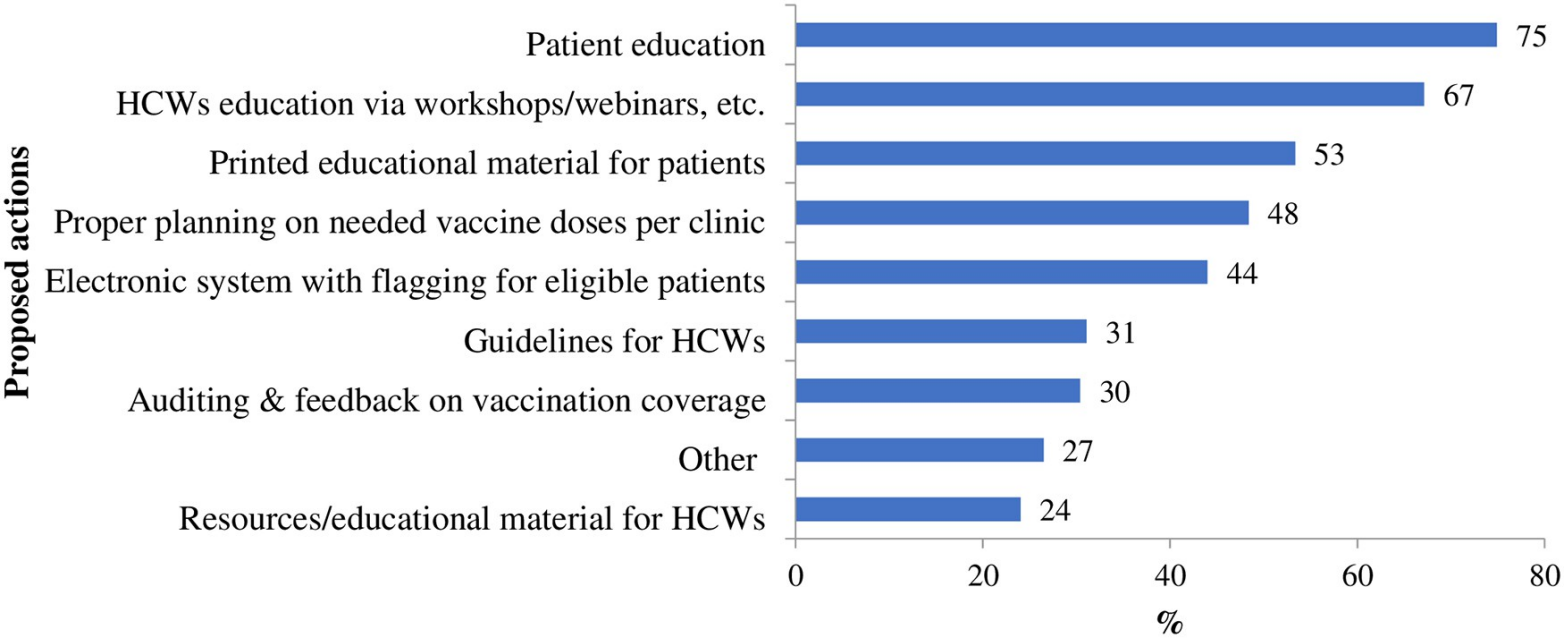
Proposed actions prior to pneumococcal vaccine (PCV) implementation by primary care doctors, Ukraine, 2021 (N = 283). HCWs: Healthcare workers.

When asked about possible actions to take prior to PCV implementation, of 223 participants, 212 (75%) proposed patient education and 190 (67%) healthcare worker education via workshops/webinars, etc. while 151 (53%) the availability of printed educational material for patients (Fig 2).

### Continuous education

**Fig 3:**
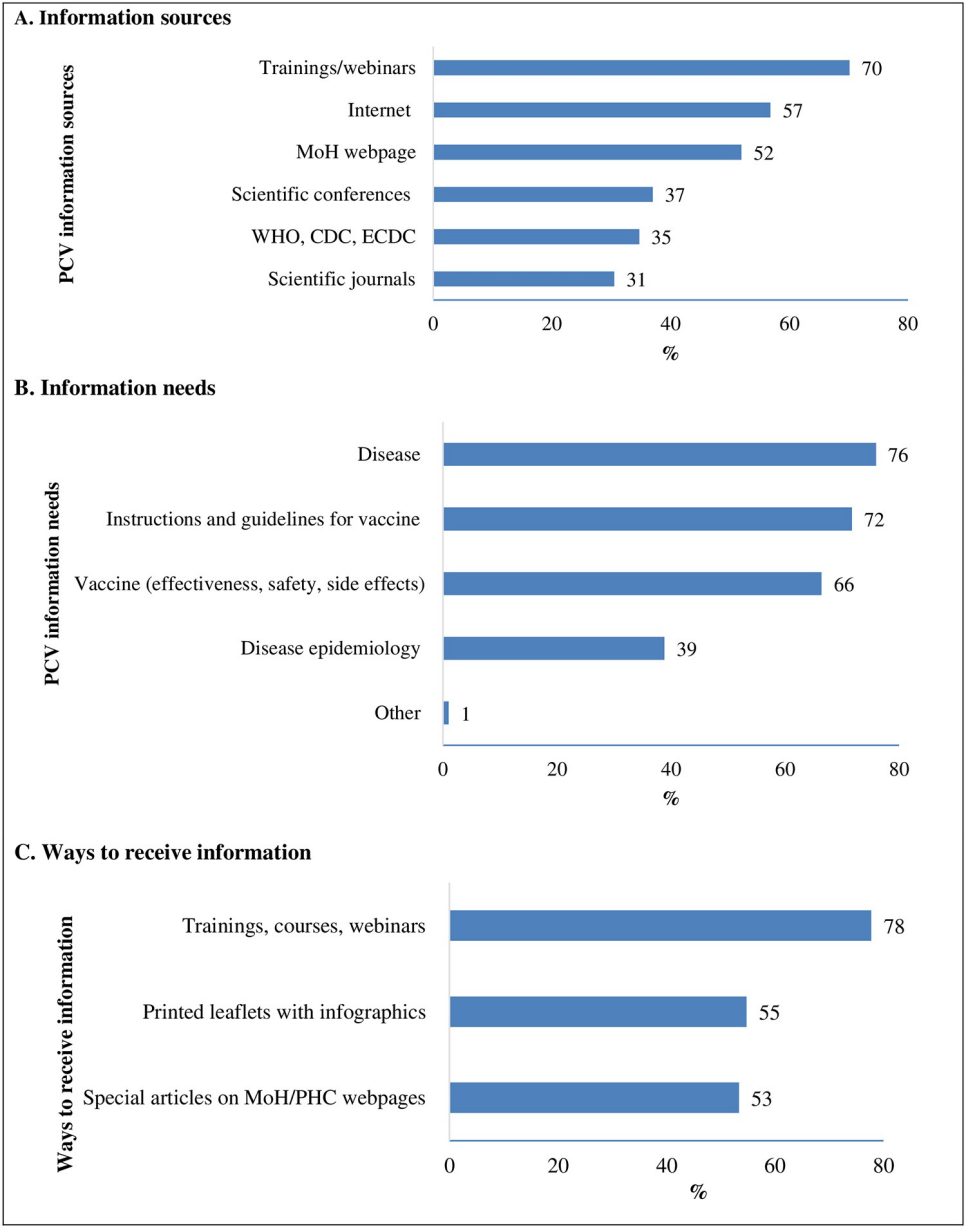
Pneumococcal vaccine (PCV) information sources, needs and ways of obtaining among primary care doctors, Ukraine, 2021 (N = 285). MoH: Ministry of Health of Ukraine; WHO: World Health Organization, CDC: Centres for Disease Control and Prevention, ECDC: European Centre for Disease Prevention and Control, PHC: Public Health Centre of the Ministry of Health of Ukraine.

Out of 283 participants, 200 (70%) reported they get information about PD and PCV via educational trainings and webinars and 162 (57%) from the internet (Fig 3A). Additionally, 215 (76%) wanted to receive information on the disease, clinical manifestations and treatment and 203 (72%) wanted to have instructions and guidelines related to PCV use, vaccination schedule, dosing, etc. (Fig 3B). The preferable way to receive information was educational trainings, courses or webinars (220/283; 78%) followed by printed leaflets with infographics (155/283; 55%) (Fig 3C).

## Discussion

In this nationwide survey, we observed moderate pneumococcal knowledge, positive PCV attitudes and neutral practices among physicians working in primary healthcare facilities with parental-related barriers being evaluated as biggest in contrast to ones related to doctors/nurses, vaccine access and logistics.

We observed moderate disease, vaccine and overall knowledge, a finding in line with other studies on vaccine preventable diseases and on pneumococcal vaccine that report physicians’ vaccine knowledge as inadequate and with gaps [18–21]. A European survey among primary care physicians highlighted that knowledge/awareness of pneumonia as the commonest presentation of pneumococcal infections was the most well-known manifestation, as did the vast majority of participants in our study however, there was a relatively low knowledge of the term invasive pneumococcal disease [22]. We found that older doctors knew less about PD, a relationship not well investigated, however a recent study found that physicians practicing longer may be at risk for providing lower-quality care [23]. Discussions among experts are ongoing on how to ensure maintenance of physicians’ clinical skills throughout their careers as older age could negatively influence disease knowledge probably due to lack of refreshing or regular educational trainings and the fact that some primary care physicians may be seeing pneumonia patients less often in their practices [24]. Our finding that doctors aware of child-related UMO, the main Ukrainian ministerial guideline regulating vaccinations and providing information and instructions, knew more about PD, is supported by a study in which guideline awareness was a factor associated with adequate knowledge assessment scores leading to a recommendation to develop innovative education strategies to publicize guidelines [25].

The vast majority of study participants had positive attitudes towards PCV effectiveness, safety and importance although we did not identify any associated factors. Other studies have also reported similar findings noting that a very high proportion of participants believed that these vaccines might reduce the seriousness of pneumococcal disease and the risk of hospitalization [20, 26, 27]. The positive attitude that influenza and pneumococcal vaccines reduce hospitalization was significantly more common in general practitioners with fewer years of professional activity, in those who dedicated longer hours per week to their professional activity, and in those who relied on scientific journals as a source of information on vaccinations [27].

Almost two thirds of study participants were recommending the vaccine to children and just below half to the elderly while female sex, positive attitudes towards PCV and identification of vaccine unavailability as medium/big barrier were all factors associated with positive practices. However, the numbers of vaccinated people in Ukraine remain very low and this may be related to the reasons for participants not recommending the vaccine, mainly its high cost and a mistrust patients have towards it [9]. Studies on PCV among healthcare workers found that almost one fifth never recommended the vaccine while patients’ refusal was a common reported barrier [11, 20]. The finding that positive attitudes towards PCV are positively associated with practice is in line with other studies among generalist and subspecialist physicians, including paediatricians, which reported that negative clinicians’ attitude was the second commonest cause of non-vaccination and that vaccine safety, efficacy, disease severity and system-related issues were important factors to recommend PCV [12, 26, 28, 29]. In our multivariate analysis, age was a confounder to positive practices—as well as to vaccine and overall knowledge and attitudes—however another study found that younger primary doctors (31–40 years) and those with relatively less experience were more likely to prescribe adult vaccines including PCV, especially in certain risk groups [30].

The vast majority of study participants evaluated parental barriers to PCV implementation as the biggest and this has previously been reported in a study in the USA in which common perceived barriers for all vaccinations were parental opposition and logistical issues [31]. Almost one third of participants reported also doctors/nurses’ related barriers including high workload and limited time and, in addition, logistics-related barriers such as interruptions in the PCV supply to the vaccination sites and vaccine non-availability, that has also been reported previously [20, 25] However, elsewhere time limitation would not affect physicians’ decision to recommend PCV [24]. In our study the cost of PCV was also reported to be a major reason for not recommending it. Other studies reported cost to be an issue and a recommendation was made to the government to consider increasing the amount of subsidy for PCV [29, 32]. Recommending PCV was associated with having previously received influenza vaccine, vaccines perceived safety and efficacy, perceived pneumococcal diseases seriousness, frequency and economic burden [26, 21].

Study participants proposed a number of actions to take prior to PCV implementation including healthcare workers’ education via workshops and webinars, patient education and printed educational material as well as the implementation of an electronic system with flagging for eligible patients. Other studies also recommend improving and promoting recall-systems, having reminders, increasing doctors’ awareness and encouraging them to consult patients routinely regarding vaccinations [11, 20, 32, 33].

Our study was subject to certain limitations. Firstly, study participants may have provided socially desirable responses for fear of potential problems at work, used external sources or asked another colleague to fill the questionnaire that may have over- or underestimated some outcome results. To avoid this, we explained in the information sheet about data anonymity, confidentiality and use, and asked participants politely not to use external sources or delegate the task to somebody else. Secondly, response entry errors during questionnaire completion could have occurred influencing the data quality. To reduce this, the majority of questions were closed-ended, an appropriate explanation on how to respond was provided and participants were asked to check for possible errors before submission. Finally, we noticed some technical issues in the creation of the online questionnaire, specifically an error in jumping sections for some participants and errors in possible answers in two questions: 1) omission of the option “3 doses” for the question on the recommended number of PCV13 doses for children aged 2–6 months and therefore we considered the answer of 2 doses as the correct one and 2) we provided the answer “somewhat” instead of “very” in the question on the importance of PCV vaccine; therefore we included the “somewhat” in the category “Low importance” while in the category “High importance” we used only the “Extremely important” answers. Despite these biases and errors, and due to the simple random sampling methodology that tends to produce representative and unbiased results using as sampling frame the most updated and comprehensive list and the relative high participation rate (46%), we believe that the study participants are representative of the of the primary health care physician population and that the study results could be generalized to the primary care physician population in Ukraine.

However, we acknowledge that we were not able to implement a stratified (by region or specialization) or a clustered random sampling methodology that could have helped account for the differences in the distribution of physicians by region or specialization or for possible clustering of providers at the department or facility level.In view of the upcoming incorporation of PCV in the childhood vaccination calendar in Ukraine, it is essential to improving vaccinating physicians’, especially of older males, pneumococcal knowledge and addressing their concerns on vaccine safety and effectiveness in order to increase PCV positive attitudes and recommendation practice, and to consider the reported barriers during the PCV implementation planning. As suggested both by the participants and a previous study [11], this can be achieved by 1) developing information material and conducting educational activities for healthcare staff using different approaches (offline/online, cascade trainings) and including the promotion and explanation of the UMO and how to deal with hesitant parents so that vaccinating physicians can effectively address parental concerns and improve vaccine uptake 2) by ensuring proper logistics organization, uninterrupted vaccine supply and the implementation of an electronic system with flagging for eligible patients and electronic reminders and 3) improving parent and patient education including the development and use of printed educational material on the importance of vaccinations and PCV in specific as a means of disease prevention. It may be beneficial to organize educational campaigns for parents including through social media to increase their knowledge and inform on the inclusion of PCV in the calendar.

To address the issue of PCV cost in target groups not included in the new vaccination calendar but for which the vaccine is recommended (i.e. the elderly) we propose to consider PCV cost reduction or a discount/subsidy or partial reimbursement.

Finally, although study participants highly rated parental barriers as major obstacles, parental knowledge, attitudes, practices and barriers towards PCV have not been assessed in Ukraine, and a KAP survey in parents of children eligible to PCV is needed to confirm this finding and further inform the PCV introduction strategy.

## Conclusions

We observed moderate pneumococcal knowledge, especially in older doctors, positive PCV attitudes and neutral practices, with females and physicians with positive attitudes reporting positive practices more often. Doctors identified as medium/big mainly parental-related barriers and reported concerns of vaccine unavailability during implementation. Proper implementation planning including undisrupted vaccine supply and educational activities for doctors and parents are needed to improve pneumococcal knowledge, introduce PCV in the calendar and address concerns in order to achieve high vaccination coverage among the target groups.

## Supporting information

S1 File Survey questionnaire. (PDF)

S2 File Minimal data set. (XLSX)
