# Supplementary material for: Knowledge, attitudes and practices survey towards pneumococcal infection and vaccination among primary health care physicians, Ukraine, 2021
Source: PLoS One. 2024 Jun 6;19(6):e0304346. doi: 10.1371/journal.pone.0304346 (PMC11156333; doi:10.1371/journal.pone.0304346)
Supplement: S1 File — (PDF) [file pone.0304346.s001.pdf]

Questionnaire on knowledge, attitude and practice towards vaccination against pneumococcal infection among doctors

Dear participant, thank you for taking part in this survey on pneumococcal infection and vaccination. The completion of this questionnaire takes approximately 15 min.

This questionnaire asks a series of questions. Please select the answer(s) you consider as correct or that reflect better your views, opinions and current practices. Please try to answer the questions as sincerely as possible without using any external sources of information such as books or online sources. Some questions may have one answer only, for others you may select more than one answers while in few you can write your own text/comments.

Your anonymity and confidentiality will be ensured at all times as we will NOT collect your IP address and other identifiable information or personal data: name, name of institution, telephone, etc.; hence, the confidentiality of your personal information will be guaranteed. It will not be able to identify individuals in the report or the other outputs of the study. The gathered data will be used only for the purposes of the present study and will not be used for any commercial purposes.

If you decide to quit the study before completing the questionnaire or not to submit the completed questionnaire the data will not be collected nor stored in any platform or device. There will be no consequences if you do not complete or do not submit the questionnaire.

You can find a copy of the information sheet on study objectives, protection of data and anonymity here.

This study has been approved by the Institutional Review Board (IRB) of PHC. For more information you can contact the IRB by email: irb@phc.org.ua

Consent

1. By submitting this questionnaire I acknowledge that I have been informed about the purposes of this study and I consent to participate in it. **Please select one answer*** *[multiple choice, compulsory question]*

A. Yes [1]

B. No [0]

Practice characteristics

2. In which region do you currently work? If you work in more than one region, please select the one you spend most of your time *[drop down list]*

3. Your place of work located is in a…? Please select one answer *[multiple choice]*

A. Rural area (under 10 000 of population) [1]

B. Urban area (over 10 000 of population) [2]

4. The healthcare facility you are working most of your time is…? Please select one answer *[multiple choice]*

A. State [1]

B. Municipal [2]

C. Private - own practice [3]

D. Private – work in private healthcare facility [4]

5. In what type of healthcare facility are you working most of your time? Please indicate the healthcare facility type you are currently working most of your time *[multiple choice]*

A. Ambulatory of general practice and family medicine [1]

B. Polyclinic [2]

C. Center of primary health care [3]

D. Clinic [4]

E. Outpatient polyclinic at the hospital [5]

F. Office of a doctor-individual entrepreneur [6]

G. Other [9]

5a. If "Other", please write here your healthcare facility type [comment box]

6. How many patients approximately does the main clinic/practice you work in serve? Please select one answer *[multiple choice]*

A. Up to 3000 patients [1]

B. 3001 to 19000 patients [2]

C. More than 19000 patients [3]

Socio-demographic data

7. What is your doctors' specialization? Please select one answer *[multiple choice]*

A. Internal medicine doctor [1]

B. General practitioner [2]

C. Paediatrician [3]

D. Other [9]

7a. If "Other", please write here your doctors' specialization [comment box]

8. How many patients approximately do you serve? Please select one answer *[multiple choice]*

A. Up to 950 patients [1]

B. 951 - 1810 patients [2]

C. More than 1810 patients [3]

9. What is your professional work experience as a doctor in full years? *[drop-down]*

10. What is your current professional category? Please select one answer *[multiple choice]*

A. No category [1]

B. First category [2]

C. Second category [3]

D. Highest category [4]

11. Do you have a scientific degree? Please select as many answers as apply *[check boxes]*

A. I do not have any degree [3]

B. PhD degree [1]

C. Doctor of Science degree [2]

12. What is your age (full years)? *[drop-down]*

13. What is your sex? *[multiple choice]*

A. Male [0]

B. Female [1]

Main questionnaire

In this section, we are going to ask you some questions regarding the pneumococcal disease, its cause, transmission, treatment, prevention. Please try to answer spontaneously and provide the answers you consider as correct. Some questions may have one answer only, for others you may select more than one answers while in few you can write your own text/comments.

Knowledge

Disease knowledge

14. What is the causative agent of pneumococcal infection? Please select one answer *[multiple choice]*

A. Bacterium [1]
B. Fungus [2]
C. Protozoa [3]
D. Virus [4]
E. I am not sure about the answer [9]

15. Which patient groups are usually affected by pneumococcal diseases? Please select as many answers as apply [*check boxes]*

A. Adults of all age-groups [1]
B. Children of all age-groups [2]
C. Elderly 65+ [3]
D. Patients with comorbidities [4]
E. Children up to 5 years [5]
F. Pregnant women [6]

16. How are pneumococcal diseases transmitted? Please select one answer *[multiple choice]*

A. Person-to-person droplets [1]
B. Direct contact [2]
C. Orally (ingestion) [3]
D. Through vectors [4]
E. I am not sure about the answer [9]

17. Which of the following are clinical manifestations of pneumococcal disease? Please select as many answers as apply *[check boxes]*

A. Liver damage [1]
B. Pneumonia [2]
C. Meningitis [3]
D. Deterioration of vision [4]
E. Otitis [5]
F. Bacteraemia [6]
G. Urinary infection [7]
H. Sinusitis [8]
I. Do not know/I am not sure [9]

18. How serious the course of pneumococcal infection can become? Please select one answer *[multiple choice]*

A. Not serious at all [1]
B. Slightly serious [2]
C. Moderately serious [3]
D. Very serious [4]
E. Extremely serious [5]

19. Is there an effective treatment for pneumococcal infection? Please select one answer *[multiple choice]*

A. Yes [1]
B. No [2]
C. I don’t know/I am not sure [9]

Vaccine knowledge

20. Is there a vaccine for the prevention of pneumococcal infection? Please select one answer *[multiple choice]*

A. Yes [1]
B. No [2]
C. I do not know/I am not sure [9]

If your answer for previous question #20 was "No" or "I do not know/I am not sure" please proceed to question #33.

21. If yes, which types of vaccine currently exist? Please select as many answers as apply *[check boxes]*

A. PCV 7 [1]
B. PCV13 [2]
C. PCV20 [3]
D. PPSV23 [4]
E. PCV40 [5]
F. I am not sure about the answer [9]

22. If yes, what is the total recommended number of vaccine doses for the following patient categories with the PCV13 vaccine? Please note that a booster dose is not included in the proposed number of doses below *[multiple choice grid]*

	1 dose	*2 doses*	*3 doses*	*I'm not sure*
A. children 2-6 months [1]			+	
B. children 7-23 months [2]		+		
C. children 24-59 months [3]	+			
D. elderly ≥65years [4]	+			

23. If yes, in your opinion, how frequent are the severe adverse events caused by the pneumococcal vaccine (PCV) in the vaccinated patients? Please select one answer *[multiple choice]*

A. Not frequent at all [5]
B. Slightly frequent [4]
C. Moderately frequent [3]
D. Very frequent [2]
E. Extremely frequent [1]

24. If yes, in Ukraine, according to MoH order #595 dated 16.09.2011, in which patient groups is the pneumococcal vaccine recommended to? Please select as many answers as apply *[check boxes]*

A. Teenagers 13-16 years [1]
B. Children of all ages [2]
C. Adults of all ages [3]
D. Patients with comorbidities [4]
E. Lactating women [5]
F. Children in closed groups (boarding schools, orphanages) [6]
G. Elderly (≥65 years), especially those living in long term care facilities [7]
H. I am not aware of the existence of the MoH order #595 [8]
I. I'm not sure of the patient's groups [9]

25. Are you aware that the MoH is going to introduce the pneumococcal vaccine (PCV) into the National Calendar of vaccinations? Please select one answer *[multiple choice]*

A. Yes [1]
B. No [2]
C. Do not know/I am not sure [9]

Attitudes

26. In your opinion, how important do you think pneumococcal vaccination (PCV) is against protection from pneumococcal disease in at-risk patients? Please select one answer *[multiple choice]*

A. Not important at all [1]
B. Slightly important [2]
C. Moderately important [3]
D. Very important [4]
E. Extremely important [5]

27. In your opinion, how effective do you think the pneumococcal vaccine (PCV) is in preventing invasive disease? Please select one answer *[multiple choice]*

A. Not effective at all [1]
B. Slightly effective [2]
C. Moderately effective [3]
D. Very effective [4]
E. Extremely effective [5]

28. In your opinion do you think the pneumococcal vaccine is safe? Please select one answer *[multiple choice]*

A. Not safe at all [1]
B. Slightly safe [2]
C. Moderately safe [3]
D. Very safe [4]
E. Extremely safe [5]
F. Depends on which country produces the pneumococcal vaccine [6]
G. I do not know [9]

29. Are you satisfied with the general information provided by the MoH about the pneumococcal vaccine (PCV) in their website? Please select one answer *[check boxes]*

A. Not satisfied at all [1]
B. Slightly satisfied [2]
C. Very satisfied [3]
D. Moderately satisfied [4]
D. Extremely satisfied [5]
E. I do not know [9]

Practices

30. Do you perform vaccinations in your everyday practice? Please select one answer *[multiple choice]*

A. Never [1]
B. Rarely [2]
C. Sometimes [3]
D. Often [4]
E. Always [5]

If your answer in previous question #30 was "Never" please proceed to the question #33.

31. Do you currently recommend vaccination against pneumococcal disease to your elderly (>65 age) patients and/or to children up to 5 years? Please select one answer *[multiple choice grid]*

	Never [1]	Rarely [2]	Sometimes [3]	Often [4]	Always [5]	I do not see these patients [6]
Children up to 5 years				+	+	
Elderly >65 age				+	+	

If your answer in previous question #31 was "Often" or "Always" or "I do not see these patients" please proceed to the question #33.

32. If you never, rarely or sometimes recommend vaccination against pneumococcal disease (PCV) to the elderly patients and/or to children up to 5 years, what are the main reasons? Please select as many answers as apply *[check boxes]*

A. My patients have a mistrust to PCV [1]
B. The vaccine is too expensive for my patients [2]
C. I do not think PCV vaccine is effective [3]
D. I do not think PCV vaccine is safe [4]
E. My patients refuse any vaccinations [5]
F. PCV is not an important vaccination for these patients [6]
G. Other [9]

32a. If "Other", please write here your reason (reasons) *[comment box]*

33. Where do you get information about pneumococcal infections and pneumococcal vaccination? Please select as many answers as apply *[check boxes]*

A. From educational trainings, webinars [1]
B. WHO, CDC, ECDC sources of information [2]
C. From the MoH official web page [3]
D. From scientific journals [4]
E. From the Internet [5]
F. From colleagues at conferences [6]
G. Other [9]

33a. If "Other", please write here your others sources of information *[comment box]*

34. What kind of information in terms of pneumococcal infections and pneumococcal vaccination would you like to receive from the authorities? Please select as many answers as apply *[check boxes]*

A. Information on the disease, clinical manifestations and treatment [1]
B. Information on PCV effectiveness, safety, side effects [2]
C. Instructions and guidelines related to PCV use, vaccination schedule, dosing [3]
D. Information on pneumococcal infections epidemiology [4]
E. Other [9]

34a. If "Other", please write here your other kind of information you would like to receive *[comment box]*

35. How would you like to receive information on pneumococcal infections and pneumococcal vaccination from the authorities? Please select as many answers as apply *[check boxes]*

A. Educational trainings, courses or webinars [1]
B. Special articles on the MoH or PHC web pages [2]
C. Printed leaflets with infographics [3]
D. Electronic leaflets with infographics [4]
E. Other [9]

35a. If "Other", please write here how you would like to receive information *[comment box]*

Possible problems/difficulties

36. Which of the listed factors can be, in your opinion, the main problems or difficulties in the implementation of vaccination against pneumococcal disease in Ukraine for children up to 5 years? Are these problems small, medium or big? Please respond for each separate possible problem/difficulty and evaluate their severity *[multiple choice grid]*

		**Please select the most appropriate answer for each of the proposed problems/difficulties and state if you consider them small, medium or big**				
	Possible problems/difficulties	***Not a problem [0]***	***Small [1]***	***Medium [2]***	***Big [3]***	***I do not know/Not applicable for me [9]***
1	Insufficient knowledge among the parents about the severity of pneumococcal infections in children [1]					
2	Lack of knowledge among the parents about the existence and benefits of PCV vaccine [2]					
3	Parents doubts about PCV effectiveness [3]					
4	Poor understanding of the real value of vaccines in general by the parents [4]					
5	Fear among the parents on the possible adverse events as a result of PCV vaccination [5]					
6	Mistrust among the parents towards certain countries that produce vaccines [6]					
7	General public rejection of all vaccinations [7]					
8	Interruptions in the supply of PCV vaccines by MoH to vaccination sites/non-availability of the vaccine [8]					
9	Long distance to the clinic/immunization site [9]					
10	Inconvenient opening hours of the clinic/immunization site [10]					
11	Doctors’ limited consultation time with patients [11]					
12	High workload of doctors and nurses/limited time to perform PCV vaccination [12]					
13	Lack of available staff to perform PCV vaccination [13]					
14	Doctors’ lack of knowledge about PCV vaccination guidelines [14]					
15	Lack of storage space/cold chain equipment to store vaccines [15]					
16	Lack of electronic system to identify and follow up eligible patients for PCV vaccination [16]					
17	Unknown prior immunization status of eligible patients [17]					

36a. Please state here any other problems/difficulties in the implementation of PCV vaccination in Ukraine that are not listed above but which you consider as important *[comment box]*

37. In your opinion which actions may help in the implementation of PCV vaccination in Ukraine? Please select as many answers as apply *[check boxes]*

A. Patient education
B. Healthcare workers (HCW) education via workshops, webinars, etc.
C. Printed educational material for patients
D. Guidelines for HCWs
E. Other useful resources and educational material for HCWs
F. Proper planning on vaccine doses needs per clinic according to eligible population
G. Proper auditing and feedback regarding vaccination coverage
H. Having an electronic system with flagging to automatically identify and follow up eligible patients
I. Other [9]

37a. If "Other", please write here other actions you think can mitigate barriers or help in the implementation of the PCV vaccine *[comment box]*

Thank you for completing the questionnaire.

For more information or any questions you may have you can contact Dr Oksana Artemchuk at tel: XX or email: XX.

Please find below some links with useful information about PCV vaccination and pneumococcal infections

https://phc.org.ua/news/vprovadzhennya-do-kalendarya-profilaktichnikh-scheplen-pnevmokokovoi-konyugovanoi-vakcini (Ukrainian)

https://www.cdc.gov/vaccines/vpd/pneumo/hcp/index.html (English)

https://www.who.int/immunization/diseases/pneumococcal/en/ (English)

SUBMIT
